# Clinical phenotype and allergen sensitization in the first 2 years as predictors of atopic disorders at age 5 years

**DOI:** 10.1186/s40413-015-0082-z

**Published:** 2015-12-02

**Authors:** Phaik Ling Quah, Evelyn Xiu Ling Loo, Gabriella Nadine Li Yuan Lee, I-Chun Kuo, Irvin Gerez, Genevieve Villablanca Llanora, Yiong Huak Chan, Marion Aw, Lynette Pei-Chi Shek, Bee Wah Lee

**Affiliations:** Department of Paediatrics, Yong Loo Lin School of Medicine, National University of Singapore, Singapore, Singapore; Singapore Institute for Clinical Sciences (SICS), Agency for Science, Technology and Research (A*STAR), Singapore, Singapore; Biostatistics Unit, Yong Loo Lin School of Medicine, National University of Singapore, Singapore, Singapore; Khoo Teck Puat-National University Children’s Medical Institute, National University Hospital, National University Health System, Singapore, Singapore; Department of Paediatrics, NUHS Tower Block, Level 12, 1E Kent Ridge Road, Singapore, 119228 Singapore

**Keywords:** Eczema, Wheeze, Rhinitis, Allergen sensitization

## Abstract

**Introduction:**

From a birth cohort of at-risk Asian infants, we prospectively investigated the role of early onset allergen sensitization and clinical phenotypes as risk factors for atopic disorders at the age of 5 years.

**Methods and materials:**

The study recruited 253 families with a history of allergic disease in a first degree relative from an antenatal clinic in Singapore. The children were followed prospectively to assess clinical outcomes and skin prick test was performed at 2 and 5 years of age.

**Results:**

Allergen sensitization (food and/or house dust mites) alone at 2 years of age was not associated with increased risk of wheeze and eczema at 5 years. However, the clinical phenotype (eczema and wheeze) with or without the presence of concomitant allergen sensitization at 2 years increased this risk. For eczema, eczema alone at year 2 increased the risk of eczema at year 5 (adjOR = 7.1; 95 % CI: 1.8–27.8) and this was further increased by the presence of allergen sensitization (adjOR = 25.4; 95 % CI: 4.7–138.5) and the concomitant presence of both wheeze and allergen sensitization (adjOR = 64.9; 95 % CI: 4.7–900.0). For wheeze, wheeze alone at 2 years (adjOR = 4.5; 95 % CI: 1.4 -14.8), and wheeze with concomitant allergen sensitization and eczema (adjOR = 13.9; 95 % CI: 1.2–168.5) increased the risk of wheeze at 5 years. The exception was rhinitis, where allergen sensitization alone at 2 years (adjOR = 5.6; 95 % CI: 1.1–29.2) increased the risk of rhinitis at 5 years. Early onset of eczema at 2 years also increased the risk of rhinitis (adjOR = 6.8; 95 % CI: 2.0–23.1).

**Conclusion:**

In this Asian birth cohort, the clinical phenotype (eczema and wheeze) with or without concomitant allergen sensitization in the first 2 years of life were strong predictors of atopic disorders at 5 years.

## Background

Although eczema, wheeze and rhinitis are considered atopic disorders, their onset in early childhood may occur in the absence of allergy or allergic sensitization [1–3]. It is well known that children with atopic disease often exhibit IgE sensitization [4], but the ability of this sensitization to predict the subsequent development of atopic disease is less clear. Thus, there has been considerable interest in understanding the relationship between early-life atopic manifestations and allergen sensitization on the outcome of atopic disorders in children. The aim was to study the relationship of the clinical phenotype and allergen sensitization in the first 2 years of life on the manifestations of eczema, wheeze and rhinitis at 5 years of age.

## Materials and methods

### Study population

This study utilized data collected prospectively from a birth cohort derived from a double-blind, placebo-controlled randomized study (ClinicalTrials.gov Identifier: NCT00318695) that was conducted to assess the effect of probiotic supplementation in the first 6 months of life on the incidence of allergic diseases. The study recruited 253 families with a history of allergic disease from the antenatal clinics at the National University Hospital, Singapore from May 2004 to June 2006. Details of subject recruitment and clinical assessment have been previously described [5]. The primary clinical outcome measure was the incidence of eczema, and the secondary outcome measures were wheeze, rhinitis and allergen sensitization. Analysis at the age of 12 months showed no statistical difference in eczema, wheeze and allergen sensitization between the probiotic supplemented group and controls [5]. Written informed consents were obtained from all families. The study was approved by the National University Hospital’s ethics review committee (DSRB Ref Code: B/00/322).

### Definition of eczema, wheeze and rhinitis and clinical follow-up

Infants were evaluated clinically by a paediatrician at 1, 2 and 5 years of age, which involved a detailed history, recording of anthropometric data and clinical examination, including looking for the presence of allergic disorders. Questionnaires (not validated) were also administered by the candidate and research nurses at these visits to record clinical disease and environmental exposures, including day care, sibship, use of antibiotics, passive smoke and pets. Biweekly phone calls were performed for the first 2 years after which once every three month phone contacts were done to collect data on the health status of the children. Eczema was defined as a pruritic rash over the face and/or extensors with a chronic relapsing course, as described by Hanifin and Rajka and modified by Seymour et al. for infants [6]. The Scoring Atopic Dermatitis (SCORAD) index was used to objectively score the severity of atopic dermatitis [7]. Recurrent wheeze/asthma was diagnosed if the child had three episodes of nocturnal cough with sleep disturbances or wheezing symptoms, separated by at least seven days, in a setting where recurrent wheeze/asthma was likely and conditions other than allergy have been excluded [8]. The subjects were diagnosed with rhinitis if the child had rhinorrhea, nasal obstruction, nasal itching and sneezing which were reversible spontaneously or with treatment and that was not due to a respiratory infection as per recommendations from the World Health Organization (WHO) Allergic Rhinitis and its Impact on Asthma workshop (ARIA) [9].

### Skin prick testing

Skin prick test was performed at 2 and 5 years using standardized technique with common allergen extracts, including soy (Alyostal, Stallergenes Laboratoires, France), cow’s milk, egg yolk, egg white, peanuts and shrimp, house dust mite (HDM) allergens - *Dermatophagoides pteronyssinus* (Greer Laboratories, Lenoir, NC) and *Blomia tropicalis* (manufactured in-house) [10]. A wheal size of at least 3 mm in diameter above the negative control was considered positive [11].

### Statistical analysis

Statistical analysis was performed using the SPSS software version 17.0 for Windows (SPSS, Inc. Chicago III, and USA). Quartiles and median cut off were generated for SCORAD scores to study the effect of eczema severity on clinical outcomes of eczema, wheeze, rhinitis, and allergen sensitization at 5 years of age. Chi-square analysis was used to compare univariate relationships between risk factors and occurrence of eczema, wheeze and rhinitis at 5 years. Logistic regression analyses were used to evaluate the independent roles of clinical phenotypes or/and allergen sensitization in the first 2 years of life with the clinical outcomes of eczema, wheeze, rhinitis and allergen sensitization at 5 years of age. The dependant variable was eczema, wheeze, rhinitis and allergen sensitization at 5 years and the covariate was a 16 level variable accounting for the risk severity of 4 variables (eczema, wheeze, rhinitis and HDM sensitization in the first 2 years of life). Confounding variables with a *p* value <0.1 were included into the logistic regression model for adjustment. These factors included: probiotic supplementation, mode of delivery, gender, serum total IgE at 1 year, ethnic group, paternal atopy, maternal atopy and sibling atopy.

## Results

**Fig. 1:**
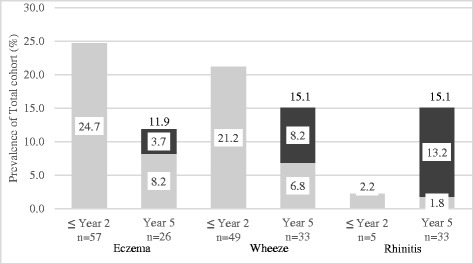
Prevalence of atopic phenotypes from birth up to 2 years and at 5 years old. Light grey columns represent % of subjects with onset 0–2 years and dark grey columns % of subjects with onset between 2 and 5 years. Prevalence is presented as percentage. The cohort comprises of 231 and 219 subjects at year 2 and 5 respectively

There were 253 at risk newborns recruited to this study. The ethnic distribution was Malay (45.5 %), Chinese (43.5 %), and others (11.1 %), with 53 % of the cohort female. At 2 years, 231 (91.3 %) subjects remained in the study, and 219 (86.5 %) were left at 5 years. There were no significant differences in demographics between those who remained in the study and the drop-outs (data not shown). The prevalence of the eczema, wheeze and rhinitis showed varying trends in the first 5 years of life (Fig. 1). For eczema, the prevalence decreased from 24.7 % (57/231) to 11.9 % (26/219) at the age of 5 years due to remission of early onset eczema. Out of the 26 subjects with eczema at year 5, 8 subjects had onset after the age of 2 years. The prevalence of wheeze was relatively stable throughout at 21.2 % (49/231) at 2 and 15.1 % (33/219) at 5 years, with 30.6 % (15/49) of early onset (<2 years) wheeze persisting to five years. Out of those with early onset wheeze, 25 (51.0 %) had recurrent wheeze. In contrast, the trends for rhinitis showed a markedly increase from 2.2 % (5/231)) at year 2 to 15.1 % (33/219) at year 5.

**Fig. 2:**
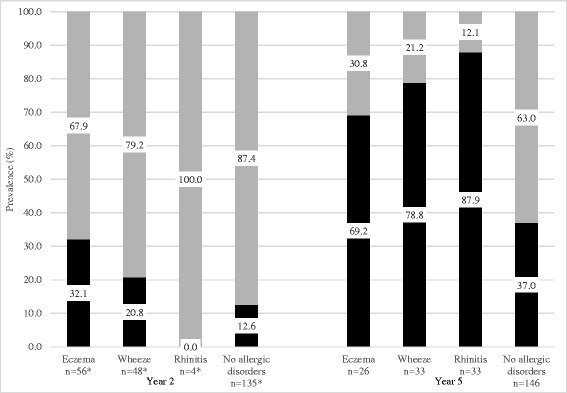
Prevalence of house dust mite sensitization in relation to clinical atopic phenotypes at 2 and 5 years old. Light grey columns represent % of non-atopic phenotype prevalence and dark grey columns represent % of atopic phenotypes prevalence. Prevalence is presented as percentage. * indicates missing 1 SPT data

In terms of allergen sensitization, house dust mite (*Dermatophagoides pteronyssinus* and *Blomia tropicalis*) sensitization was more common than sensitization to common foods (cow’s milk, egg, soy, shrimp and peanut) at 2 years old (*p* < 0.05) (Table 1). Positive skin prick test responses to house dust mites increased further from 17.0 % (39/229) at year 2 to 50.7 % (111/219) at 5 years. Sensitization to food allergens at the age of 2 years was relatively low, with 2.2 % (5/229) sensitized to egg white, 0.4 % (1/229) each to egg yolk, cow’s milk and soy, 1.4 % (3/222) each to peanut and shrimp allergens. Of those sensitized to food allergens, only one subject in the cohort had clinical allergy to egg. There was no significant association between eczema severity as measured by SCORAD in the first 2 years of life and allergen sensitization at 2 years (Table 2). The prevalence of clinical phenotypes associated with HDM sensitization (at least one HDM allergen) was comparatively low in early life but increased considerably at 5 years (Fig. 2). In eczema subjects, HDM sensitization increased from 32.1 % (18/56) at year 2 to 69.2 % (18/26) at year 5, and similarly in wheeze subjects from 20.8 % (10/48) at year 2 to 78.8 % (26/33) at year 5. The rate of HDM sensitization was 87.9 % (29/33) in rhinitis subjects at year 5.

**Table 1:** Characteristics of Allergen Sensitization at 2 Years and at 5 Years of Age

	2 years old	5 years old
Allergen sensitization (%)	*n* = 229 ^a^	*n* = 219
Food allergen (any)	7 (3.1 %) *	N.D.
Cow’s milk	1 (0.4 %)	
Egg white	5 (2.2 %)	
Egg Yolk	1 (0.4 %)	
Soy	1 (0.4 %)	
Shrimp	3 (1.4 %) +	
Peanut	3 (1.4 %) +	
Inhalant allergens (any dust mite)	39 (17.0 %) *	111 (50.7 %)
*Dermatophagoides pteronyssinus*	37 (16.2 %)	97 (44.3 %)
*Blomia tropicalis*	11 (4.8 %)	81 (36.5 %)

*N.D.* not done

^a^ indicates missing SPT data, * indicates statistically significant (*p* < 0.05), + indicates *n* = 222 instead of 229

**Table 2:** Characteristics of allergen sensitization at 2 years of age against severity of Eczema up to 2 years

	Allergen sensitization		
SCORAD^b^ (quartile range)	Presence	Absence	Total
Upper quartile (<22.56)	8	5	13
Mid upper quartile (17.11–22.55)	4	10	14
Mid lower quartile (11.075–17.10)	3	10	13
Lower quartile (<11.075)	6	7	13
Total quartile	21	32	53^a^

^a^There were 4 subjects with missing SCORAD data

^b^ Denotes highest SCORAD recorded in the first 2 years of life

Notably, we observed transition from a non-sensitized phenotype at 2 years to a sensitized phenotype in 8/18 subjects whose eczema persisted to 5 years, and in 10/15 subjects whose wheeze persisted to 5 years.

To examine the role of HDM sensitization and clinical phenotypes in the first 2 years of life on the development of eczema, wheeze, rhinitis and allergen sensitization at year 5, a 16-level variable was formed using these 4 variables to account for their risk severity by placing all the combinations of the 4 variables into one 16 level variable (Table 3). Similar results were also obtained when any allergen sensitization (both HDM and food) was analysed (data not shown). We found that having eczema at year 2 is a significant risk factor for eczema at year 5 (adjOR = 7.1; 95 % CI = 1.8–27.8). In addition, the presence of concomitant allergen sensitization alone or allergen sensitization with wheeze further increased the risk (adjOR = 25.4; 95 % CI = 4.7–138.5, and adjOR = 64.9; 95 % CI = 4.7–900.0, respectively) (Table 3).

**Table 3:** Clinical phenotypes and allergen sensitization at 2 years as risk factors for atopic outcomes at 5 years

Allergic phenotypes		
5 years	Phenotype risk factors at 2 years	Adjusted OR (95 % CI) **
Eczema	No eczema, wheeze, rhinitis and allergen sensitization (*n* = 110) ^a^	1
*n* = 26	Allergen sensitization only (*n* = 16)	4.0 (0.6–27.5)
	**Eczema only (** ***n*** **= 32)** *	**7.1 (1.8–27.8)**
	Wheeze only (*n* = 32)	0.7 (0.1–7.2)
	**Eczema and allergen sensitization (** ***n*** **= 12)** *	**25.4 (4.7–138.5)**
	Wheeze and allergen sensitization (*n* = 4)	9.6 (0.6–142.4)
	Eczema and wheeze (*n* = 3)	28.3 (1.0–814.9)
	**Eczema, wheeze and allergen sensitization (** ***n*** **= 5)** *	**64.9 (4.7–900.0)**
Wheeze	No eczema, wheeze, rhinitis and allergen sensitization (*n* = 110) ^a^	1
*n* = 32 ^b^	Allergen sensitization only (*n* = 16)	1.9 (0.3–10.4)
	Eczema only (*n* = 32)	1.4 (0.4–5.5)
	**Wheeze only (** ***n*** **= 32)**	**4.5 (1.4–14.8)**
	Eczema and allergen sensitization (*n* = 12)	3.1 (0.6–15.7)
	Wheeze and allergen sensitization (*n* = 4)	7.8 (0.8–71.2)
	Eczema and wheeze (*n* = 3)	20.9 (0.9–499.6)
	Eczema, wheeze and allergen sensitization (*n* = 5)	13.9 (1.2–168.5)
	Eczema, rhinitis and allergen sensitization (*n* = 2)	16.5 (0.8–360.2)
Rhinitis	No eczema, wheeze, rhinitis and allergen sensitization (*n* = 110) ^a^	1
*n* = 32 ^b^	**Allergen sensitization only (** ***n*** **= 16)**	**5.6 (1.1–29.2)**
	**Eczema only (** ***n*** **= 32)** *	**6.8 (2.0–23.1)**
	Wheeze only (*n* = 32)	1.7 (0.4–7.9)
	Eczema and allergen sensitization (*n* = 12)	3.8 (0.7–20.6)
	Eczema, wheeze and allergen sensitization (*n* = 5)	10.1 (1.0–104.1)
Allergen sensitization	No eczema, wheeze, rhinitis and allergen sensitization (*n* = 110) ^a^	
*n* = 111	**Allergen sensitization only (** ***n*** **= 16)** *	**9.0 (2.3–35.3)**
	**Eczema only (** ***n*** **= 32)** *	**3.6 (1.5–8.7)**
	Wheeze only (*n* = 32)	1.1 (0.4–2.7)

Statistically significant variables (*p* < 0.05) highlighted in bold and (*p* < 0.01) variables highlighted in bold and marked with *

** indicates adjusted for other variables (such as probiotic supplementation, gender, ethnic group, mode of delivery, maternal atopy, paternal atopy, sibling atopy and IgE measurements at 1 year) in the table that were selected based on the univariate analysis and a *p* value of <0.1

^a^ indicates the reference group

^b^ indicates missing SPT

For wheeze at 5 years of age, wheeze alone at 2 years was a risk factor (adjOR = 4.5; 95 % CI = 1.4–14.8) (Table 3). The concomitant presence of eczema and allergen sensitization (adjOR = 13.9; 95 % CI: 1.2–168.5) increased the risk of wheeze at 5 years. When wheeze at 2 years were subgrouped into recurrent wheeze and single episode wheeze, both groups showed similar risks for wheeze at 5 years (data not shown).

For rhinitis at 5 years of age, early allergen sensitization to house dust mites increased the risk of rhinitis at 5 years (adjOR = 5.6; 1.1–29.2). Early onset of eczema by 2 years also increased the risk of rhinitis (adjOR = 6.8; 95 % CI = 2.0–23.1).

For HDM allergen sensitization at 5 years of age, allergen sensitization at 2 years was the strongest risk factor (adjOR = 9.0; 95 % CI = 2.3–35.3), but early onset of eczema also increased the risk (adjOR = 3.6; 95 % CI = 1.5–8.7).

The effect of eczema severity on atopic outcomes at 5 years was evaluated while adjusting for other variables using logistic regression. We found that although both groups (SCORAD above and below median) were significantly associated with eczema at 5 years, however, those with more severe eczema at the age of 2 years (SCORAD above median for group) was more strongly associated with eczema at year 5 than those with less severe eczema (SCORAD below median for group) (SCORAD above median: adjOR =15.2; 95 % CI = 4.3–53.7; SCORAD below median: adjOR = 5.7; 95 % CI = 1.4–22.7). Only those with more severe eczema was significantly associated with the development of allergen sensitization at 5 years (SCORAD above median: adjOR = 2.9; 95 % CI = 1.0–8.5). Eczema severity at the age of 2 years did not affect outcomes for wheeze and rhinitis at 5 years (*p* > 0.05).

## Discussion

This study aimed to look at the role of early onset allergen sensitization and clinical phenotypes of eczema and wheeze at the age of 2 years as predictors of the atopic outcomes at the age of 5 years. This Asian at-risk for allergy birth cohort has shown that allergen sensitization alone at 2 years of life was not a risk factor for eczema and wheeze at 5 years old. Instead, the clinical phenotype alone (ie, eczema and wheeze) were risk factors, and the concomitant presence of allergen sensitization further increased this risk. Therefore, early onset eczema and wheeze before 2 years of age, are more likely to persist till the age of 5 years old. The allergen panel chosen were common food allergens in our population [12], and dust mite allergens. House dust mites (*Dermatophagoides pteronyssinus, Blomia tropicalis*) are the most important inhalant allergen in tropical Singapore as has been shown in a population study of atopic teenagers and young adults, where almost all subjects were found to be exclusively sensitized to house dust mite allergens [13]. In a study by Chew et al*.*, similar results were found in atopic children where >90 % of the subjects were sensitized to at least one of the 2 dust mites [14].

Other studies had similar observations but mainly with the eczema phenotype. The German Multicentre Atopy Study (GMAS) that recruited 1314 infants [15] observed that children with early onset eczema and concomitant wheeze were 3 times more likely to have wheeze at 7 years compared to children without early onset eczema Further, two birth studies from western Sweden as well as Germany (GMAS) reported early onset eczema as risk factors for allergic rhinitis in later childhood [16, 17]. In The Prevention of Allergy among Children in Trondheim (PACT) study, children with eczema at 2 years were 2 times more likely to develop asthma at 6 years [18]. The Tuscon Children’s Respiratory Study also found early onset of eczema in the first year of life was independently associated with persistent wheezing at 6 years of life [2]. Similarly, the Dampness in Building and Health Study that recruited 3124 children aged 1–2 years and followed them for 5 years reported that children with early onset eczema had increased odds of developing asthma and rhinitis [19].

With regards to early wheeze as a risk factor, a birth cohort study from Stockholm, Sweden involving 3251 children reported that children who wheezed in the first 2 years of life were 4 times more likely to develop asthma at 8 years of age compared to the non-wheezers [20]. In addition, recurrent wheeze of more than 3 episodes in the first 2 years of life conferred additional risk to the development of asthma at the age of 8 years [20]. Similarly, the Tuscon Children’s respiratory study reported that persistent wheezing before the age of 3 years was independently associated with asthma at the age of 22 years [21]. Taken together, these observations indicate that the clinical phenotype even without allergen sensitization are risk factors for atopic disorders in later childhood, and suggests that factors other than allergy play a role in the development of allergic disorders. This postulate is substantiated by the large European population based cohort study, MeDALL (Mechanisms of the Development of ALLergy), which showed that eczema, rhinitis and asthma may coexist in the absence of IgE sensitization, and that IgE sensitization was not the dominant causal mechanism of comorbidity for these disorders ([22]).

We also observed that the clinical phenotype may precede the development of allergen sensitization in some subjects with eczema and wheeze. This is similar to a prospective Thai study which showed that children with non-allergic rhinitis could develop allergic rhinitis with subsequent follow up [23]. Further, the Melbourne Atopy Cohort Study in Australia also showed that children with eczema in early life were more likely to have new allergen sensitization at 1 year and 2 years [24]. We observed that the severity of eczema in the first 2 years was associated with eczema and allergen sensitization at 5 years of age. Similarly, the Consortium of Food Allergy Research Observational Study reported that children with history of atopic dermatitis had higher peanut sensitization when exposed to peanut and the sensitization rates was further increased in those with severe atopic dermatitis [25, 26]. Other studies reported the association between eczema severity and asthma [29, 30], which we did not observe in our cohort.

Although allergen sensitization may not be the dominant factor in the development of eczema and wheeze in later childhood, allergen sensitization did play a role. We observed that early onset allergen sensitization in the presence of the clinical phenotype was a cofactor in increasing the risk of subsequent atopic disease. Other studies have made similar observations. The importance of HDM in increasing the odds of development of atopic outcomes in the presence of eczema was also reported by the German Infant Nutritional Intervention (GINI) Study [31, 32]. This study showed that early HDM sensitization was a risk factor for eczema at age 6 years but only if the children already had eczema within the first 12 months [32]. In the German Multicenter Atopy Study (GMAS) birth cohort study, children with eczema, concomitant wheeze and allergen sensitization had higher risk for the development of wheeze at 7 years compared to just eczema and wheeze alone [15]. In another cohort of at-risk children from Western Australia, infants that wheezed and were sensitized to allergens in the first year of life had the highest rates of subsequent asthma development [33].

The importance of allergen (HDM sensitization) as an independent risk factor of subsequent atopy, was only seen with rhinitis in our Singapore cohort. Similar to our observations where HDM sensitization was associated with the subsequent development of rhinitis, the GINI study has showed that sensitization to aeroallergen was an independent risk factor for the development of rhinitis at 6 years of age [32]. In contrast to our findings, other studies have also shown that aeroallergen sensitization is an independent risk factor for subsequent asthma. The Tucson epidemiological study of airways obstructive disease also found that those with early sensitization to any aeroallergen before the age of 8 years were more likely to develop wheeze after the age of 8 [34]. In addition, the Melbourne Atopy Cohort Study in Australia also showed that house dust mite sensitization at the age of 1 and 2 years was associated with increased odds of development of wheeze at 12 years [35].

Of interest are the observations that eczema without allergen sensitization at the age of 2 years was a risk factor for allergen sensitization at the age of 5 years, indicating that the eczema phenotype may precede and predispose to aeroallergen sensitization. This is in support of the notion that eczema increases the risk of allergen sensitization possibly directly through the inflamed skin and predisposes to food allergy ([25]). Eczema therefore appears to be the first manifestation of atopy as defined by the atopic march. Other studies have reported similar observations. A Swedish study that recruited 94 children with eczema and followed them till 7 years of age found that early onset eczema increased the odds of subsequent development of allergen sensitization [26]. Similarly, early onset persistent eczema was significantly associated with allergen sensitization at 7 years in a high risk cohort of 373 Canadian infants [36]. In addition, a birth cohort study conducted in Isle of Wright that followed 1218 children till the age of 4 years reported that eczema in the first 2 years of life was a strong predictor for aeroallergen sensitization at 4 years [37].

These findings from cohort studies including ours support the concept of the atopic march where eczema in early life predisposes to allergen sensitization and atopic disorders in later childhood [38, 39]. Further, the airway inflammation associated with early viral wheeze has been postulated to play a role in subsequent asthma development [40].

The limitations of our study are that due to the small number of subjects with the exposure and outcomes, the confidence intervals are wide and this also reduces our statistical power to determine relationships. In addition, it is possible that wheeze at 5 years of age may not equate to persistent childhood asthma in all subjects. A longer follow up will be necessary to determine this. However, this study has documented these risk factors for a birth cohort living in the tropics. We have also documented that clinical phenotype may precede allergen sensitization.

In conclusion, although a relatively small birth cohort, our data supports the notion that both early manifestations of the clinical phenotype with or without concomitant allergen sensitization are risk factors for subsequent atopic disorders, and that the early clinical phenotypes may precede the allergen sensitization and atopic disease later in life.

## Abbreviations

CI: confidence intervals
HDM: house dust mite
OR: odds ratio
